# Efficacy of endoscopic removal of anterior malleolar ligament calcification combined with tympanic membrane repair for the treatment of conductive hearing loss

**DOI:** 10.12669/pjms.42.6.15718

**Published:** 2026-06

**Authors:** Lei Yue, Juan Li, Yue Deng, Song Shi

**Affiliations:** 1 Lei Yue, Department of Otolaryngology, Tongren Hospital, Shanghai Jiao Tong University School of Medicine, Shanghai 200336, P.R. China; 2 Juan Li, Department of Otolaryngology, Tongren Hospital, Shanghai Jiao Tong University School of Medicine, Shanghai 200336, P.R. China; 3 Yue Deng, Department of Otolaryngology, Tongren Hospital, Shanghai Jiao Tong University School of Medicine, Shanghai 200336, P.R. China; 4 Song Shi, Department of Otolaryngology, Tongren Hospital, Shanghai Jiao Tong University School of Medicine, Shanghai 200336, P.R. China

**Keywords:** Anterior malleolar ligament, Calcification, Conductive hearing loss, Endoscopic, Tympanic membrane repair

## Abstract

**Objective:**

Anterior malleus ligament calcification (AMLC) can restrict malleolar mobility, thereby impairing sound conduction through the ossicular chain and potentially contributing to conductive hearing loss (CHL). The study aimed to investigate the pathogenic role of calcification of the anterior malleus ligament in CHL and to evaluate the efficacy of endoscopic removal of AMLC with concurrent tympanic membrane repair.

**Methodology:**

Clinical data of 68 CHL patients (68 ears) who underwent endoscopic tympanoplasty in Tongren Hospital, Shanghai Jiao Tong University School of Medicine (January, 2019 – December, 2023) were retrospectively analyzed. All patients had intraoperatively confirmed AMLC and no other obvious ossicular chain lesions. Endoscopic removal of AMLC and concurrent tympanic membrane repair were performed. Pure-tone audiometry indices (air conduction threshold [ACT], bone conduction threshold [BCT], air-bone gap [ABG]) and tympanic membrane healing were compared preoperatively and at one, three and six months postoperatively to assess surgical efficacy and complications.

**Results:**

All patients were followed up for 6~12 months, with 100% primary tympanic membrane healing. Postoperative ACT and ABG were significantly improved compared to preoperative values (P<0.05). At 6 months postoperatively, mean ACT decreased from 56.32±8.75 to 25.16±5.38 dB HL, and mean ABG narrowed from 31.25±6.42 to 8.67±3.15 dB HL (both P<0.01). No severe complications occurred.

**Conclusions:**

Calcification of AML is an important cause of CHL. Endoscopic AMLC removal combined with tympanic membrane repair was associated with favorable short-term hearing improvement, with the advantages of minimal invasiveness, clear visualization, and few complications. Furthermore, for patients with CHL and intact ossicular chains, the possibility of AMLC should be vigilantly considered. However, the findings mainly reflect short-term outcomes, and the diagnosis relied heavily on intraoperative identification. Therefore, the findings should be interpreted with caution.

## INTRODUCTION

Conductive hearing loss (CHL) is common in otorhinolaryngology and is often associated with external auditory canal obstruction, tympanic membrane lesions, and ossicular chain abnormalities.^1^ Primary pathogenic factors of ossicular chain disorders include morphological anomalies or discontinuities of the malleus, incus, and stapes, such as incudomalleolar joint dislocation and stapes fixation.^2^ However, a subset of patients with CHL exhibits intact ossicular chain continuity yet suboptimal hearing improvement, suggesting the involvement of other underrecognized etiological factors.

The anterior malleolar ligament (AML) is a critical structure connecting the neck of the malleus to the tympanic part of the temporal bone, with a primary function of restricting excessive movement of the malleus and maintaining normal mechanical conduction of the ossicular chain.^3^ In recent years, with the advancement and widespread clinical application of otoscopic technology, intraoperative visualization of subtle middle ear structures has become increasingly precise, leading to growing attention on pathological changes of the AML.^4^ AML calcification (AMLC) is the hardening of the ligament connecting the malleus head to the anterior epitympanic wall. Studies have demonstrated that AMLC can restrict malleolar mobility, thereby impairing sound conduction through the ossicular chain and potentially contributing to CHL.^5^,^6^ Nevertheless, research on the correlation between AMLC and hearing loss remains limited, and no consensus has been reached regarding its clinical characteristics, diagnostic methods, and treatment strategies.

Endoscopic tympanoplasty has evolved into one of the mainstream surgical modalities for treating tympanic membrane perforation and CHL, due to its advantages of minimal invasiveness, clear surgical field of view, and rapid postoperative recovery.^7^ This retrospective study analyzed the clinical data of 68 patients with CHL who were intraoperatively confirmed to have AMLC. The study aimed to explore the pathogenic mechanism of AMLC and evaluate the therapeutic efficacy of endoscopic calcification removal combined with tympanic membrane repair, thereby providing evidence-based references for clinical diagnosis and treatment.

## METHODOLOGY

Clinical data from 68 patients (68 ears) with CHL who underwent endoscopic tympanoplasty at Tongren Hospital, Shanghai Jiao Tong University School of Medicine, from January 2019 to December 2023 were retrospectively analyzed

### Ethical approval:

This study was approved by the institutional Ethics Committee (Ref. K2024-015-01; Date: April 9, 2024). All patients presented with unilateral involvement, including 32 left-ear cases and 36 right-ear cases.

All patients completed the following preoperative examinations:

### Otoscopy:

Location, size, and margin of tympanic membrane perforation were observed, and the status of external auditory canal skin and tympanic mucosa was evaluated.

### Pure tone audiometry:

Tests were performed using a GSI 61 audiometer (Grason-Stadler Inc., USA) in accordance with GB/T 16403-1996. Air conduction thresholds (ACT) and bone conduction thresholds (BCT) at 0.5, 1, 2, and 4 kHz were recorded, and the air-bone gap (ABG) was calculated. *Tympanometry*. A Madsen Zodiac 901 tympanometer (MADSEN, Denmark) was used to detect tympanogram types and stapedial reflexes.

### High-resolution Temporal Bone Computed Tomography (HRCT):

Scans were conducted with a Siemens SOMATOM Definition Flash CT scanner in axial and coronal planes, with a slice thickness of 0.625 mm. The structures, including the middle ear cavity, ossicular chain, and mastoid air cells, were evaluated, with special attention paid to the presence of calcification shadows in the region of the AML.

### Inclusion Criteria:

- Meeting the diagnostic criteria for CHL, with pure tone audiometry showing an air-bone gap ≥ 15 dB HL and no clinically significant sensorineural hearing loss.
- Presenting with tympanic membrane perforation (central or marginal), and excluding ossicular chain discontinuity, cholesteatoma, middle ear tumors, and other lesions via otoscopy and temporal bone computed tomography (CT) scans.
- Confirmed AMLC identified intraoperatively by endoscopic visualization.
- Obtained informed consent from patients and their family members, with full cooperation in completing postoperative follow-up.

### Exclusion Criteria:

- Complicated with inner ear lesions (such as, sensorineural hearing loss, Meniere’s disease, inner ear malformation).
- Obvious ossicular chain damage (such as, malleolar handle fracture, incus long process defect).
- Congenital ossicular chain malformation.
- A history of prior middle ear surgery.
- Middle ear fungal infection.
- Severe systemic diseases (such as, diabetes mellitus, coagulation dysfunction).
- Incomplete follow-up data.

### Surgical procedure:

**Fig.1:**
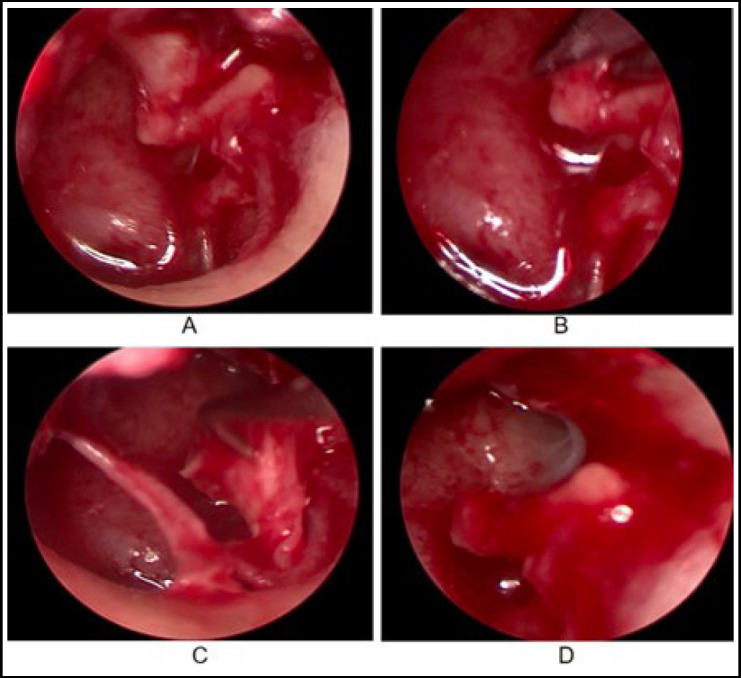
Endoscopic removal of AMLC. A. Exposure of AMLC; B. Dissection of the calcified tissue from the malleus using microscissors; C. Extraction of the calcified lesion with a hook; D. Complete removal of the calcified lesion.

All surgeries were performed by the same surgeon under general anesthesia. Patients were placed in the supine position with their heads turned to the contralateral side. The surgical procedures were as follows (Fig.1):

### Elevation of the external auditory canal skin flap to access the middle ear cavity:

A 3- mm diameter, 0° or 30° otoscope (Karl Storz, Germany) was used to enter through the natural external auditory canal. The posterosuperior wall of the external auditory canal was infiltrated with 1:10000 epinephrine for local anesthesia. An incision was made on the external auditory canal skin 6–8 mm away from the tympanic annulus in the 10 o’clock to 6 o’clock direction, extending down to the bony wall. The external auditory canal skin flap was elevated anteriorly, with the chorda tympani nerve well protected. The tympanic annulus was lifted to gain access to the middle ear cavity.

### Identification of AMLC:

Under the clear visual field provided by the endoscope, the area anterior to the neck of the malleus was examined. The AMLC presented as a white, hard, cord-like structure with clear boundaries from surrounding tissues, often accompanied by restricted malleus mobility. The restriction of AML function was assessed intraoperatively using a standardized probing maneuver. Careful differentiation from the medial chorda tympani nerve, chronic inflammatory scarring, or fibrous adhesion was required. The medial chorda tympani nerve appeared pinkish with visible vascular markings on its surface. Chronic inflammatory scarring or fibrous adhesion appear soft, whitish, and presenting as fibrous bands. AMLC was classified into three grades according to the severity of calcification:^8^

### Grade-I (Mild):

Calcification was confined to local areas of the ligament, presenting as punctate or small patchy lesions, with mild limitation of malleus mobility.

### Grade-II (Moderate):

Calcification involved the entire length of the ligament, appearing as hard cord-like lesions, with obvious limitation of malleus mobility.

### Grade-III (Severe):

Calcification extended beyond the ligament itself to the surrounding bony structures, resulting in bony fusion and almost complete loss of malleus mobility.

### Removal of calcified lesions:

A micro dissector or small hook was used to carefully dissect the edges of the calcified ligament, avoiding damage to the malleus, residual tympanic membrane margins, and tympanic mucosa. For calcified foci tightly adhering to the tympanic annulus, after dissecting the space between the lesion and the malleus neck, a miniature diamond burr (0.5 mm diameter) was applied at low speed for grinding removal, with care taken not to touch the ossicles. The procedure was terminated when the malleus mobility returned to normal (intraoperatively verified by gently probing the malleus head and observing its range of motion).

### Tympanic membrane repair:

The repair material was selected based on the size of the tympanic membrane perforation. For perforations with a diameter < 3 mm, the intracavitary implantation of tragus perichondrium (parotid side) was adopted. For perforations≥ 3 mm in diameter, a tragal cartilage-perichondrium composite graft was used. The graft material was spread flat to cover the perforation area and closely opposed to the residual margins of the tympanic membrane.

### Post-grafting surgical cavity management:

For repairs using tragal perichondrium alone, the surgical cavity was packed with gelatin sponge. For repairs using the tragal cartilage-perichondrium composite graft, no gelatin sponge was placed medial to the tympanic membrane graft. The external auditory canal was packed with gelatin sponge particles soaked in levofloxacin, dexamethasone, and growth factor, which were gently compressed for fixation. The incision on the tragus was then sutured.

### Postoperative Management and Follow-up:

- Intravenous antibiotics were administered for 1–3 days postoperatively to prevent infection.
- The sutures at the tragus were removed on the 7th postoperative day, and the gelatin sponge in the external auditory canal was debrided under otoscopy two weeks postoperatively.
- Follow-up assessments were conducted at one, three and six months postoperatively. The items assessed included taste disturbance, transient tinnitus fluctuation, postoperative vertigo or imbalance, local granulation, anterior tympanic adhesion, impaired anterior graft healing, and changes in middle ear aeration. Otoscopy (to evaluate tympanic membrane healing) and pure tone audiometry (to assess hearing improvement) were performed at each follow-up visit.

### Efficacy Evaluation Criteria:

The following indices were used to assess treatment efficacy:

### Tympanic Membrane Healing:

Otoscopy findings indicated complete closure of the tympanic membrane perforation, with a regular morphology, smooth surface, and no obvious retraction or protrusion.

### Hearing Improvement:

Pure-tone audiometry results at 6 months postoperatively were used as the primary evaluation index. Hearing success was defined as an ABG ≤ 20 dB HL or a reduction in ABG of ≥ 15 dB HL from the preoperative level.^9^

### Complications:

The occurrence of postoperative complications was recorded, including infection, facial paralysis, sensorineural hearing loss, and iatrogenic cholesteatoma.

### Statistical analysis:

All analyses were performed using SPSS 26.0 software. Measurement data were expressed as mean ± standard deviation (SD). Comparisons of hearing indicators between preoperative and postoperative time points were conducted using the paired t-test. One-way analysis of variance (ANOVA) was used to assess the statistical significance of differences in continuous variables across subgroups with varying degrees of calcification, and the least significant difference (LSD) method was used for post hoc pairwise comparisons. Repeated measures ANOVA was used for comparison of pure tone audiometry results at baseline and at 1, 3, and 6 months after surgery. Count data were presented as rates (%), and comparisons were analyzed using the Chi-square test (χ^2^ test). P < 0.05 was considered statistically significant.

## RESULTS

**Fig.2:**
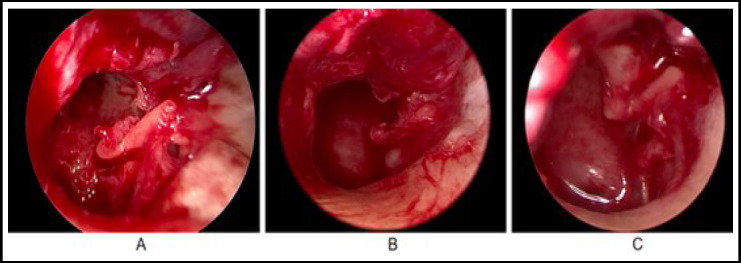
Calcification of the AML. A, Grade-I; B, Grade-II; C, Grade-III.

A total of 68 patients (38 males [55.9%] and 30 females [44.1%]) were included in the study. Patients were aged 18–65 years with a mean age of 41.2±10.5 years. The disease duration ranged from six months to 15 years, with a mean duration of 4.2±2.1 years. The clinical characteristics, preoperative examinations, and intraoperative findings of the patients are shown in Table-I. The mean preoperative ACT and BCT were 56.32±8.75 dB HL and 25.07±5.12 dB HL, respectively. The mean ABG was 31.25±6.42 dB HL. No acoustic admittance was recorded in all patients before surgery. Of 68 patients, 42 (61.8%) showed punctate or cord-like hyperdense shadows (CT value > 200 HU) in the AML area on CT scans, indicating calcification. In the remaining 26 cases (38.2%), no obvious calcification shadows were detected on CT, and calcification was confirmed only intraoperatively by otoscopy. There were 22 cases (32.4%) of Grade-I AMLC, 36 cases (52.9%) of Grade-II AMLC, and 10 cases (14.7%) of Grade-III AMLC (Fig.2). Intraoperative observations identified 28 cases (41.2%) with tympanic mucosal thickening and 12 cases (17.6%) with mild osteoproliferation of the long process of the incus. However, no severe pathological changes such as ossicular chain discontinuity or stapes fixation were detected in any of the patients.

**Table-I:** Clinical Characteristics, Preoperative Examinations and Intraoperative Findings of 68 Patients.

Category	Subcategory	Case Number (n=68)	Proportion
Baseline Characteristics	Male (yes)	38	55.9%
	Age (years)	41.2±10.5	-
	Disease Course (years)	4.2±2.1	-
Etiology	Chronic Suppurative Otitis Media	52	76.5%
	Traumatic Tympanic Membrane Perforation	12	17.6%
	Congenital Tympanic Membrane Perforation	4	5.9%
Tympanic Membrane Perforation Site	Central Perforation of Pars Tensa	56	82.4%
	Marginal Perforation of Pars Tensa	12	17.6%
Tympanic Membrane Perforation Diameter	< 3 mm	28	41.2%
	3–5 mm	32	47.1%
	> 5 mm	8	11.8%
Preoperative Pure Tone Audiometry	ACT (dB HL)	56.32±8.75	-
	BCT (dB HL)	25.07±5.12	-
	ABG (dB HL)	31.25±6.42	-
Preoperative Tympanometry	Result	No acoustic admittance recorded	-
Preoperative Temporal Bone CT	Calcification in AML Area (CT value > 200 HU)	42	61.8%
	No Obvious Calcification Shadow on CT (Confirmed Intraoperatively)	26	38.2%
Intraoperative AMLC Grade	Grade-I (Mild) - Local punctate/small patchy calcification, mild limitation of malleus mobility	22	32.4%
	Grade-II (Moderate) - Diffuse cord-like calcification involving the entire ligament, obvious limitation of malleus mobility	36	52.9%
	Grade-III (Severe) - Calcification extending beyond the ligament, bony fusion with surrounding structures, almost complete loss of malleus mobility	10	14.7%
Intraoperative Comorbidities	Tympanic Mucosal Thickening	28	41.2%
	Mild Osteoproliferation of Long Process of Incus	12	17.6%
	Ossicular Chain Discontinuity/Stapes Fixation	0	0%

At one month postoperatively, the tympanic membrane achieved complete healing in 62 patients (91.2%). By three months postoperatively, the remaining six patients (8.8%) also reported tympanic membrane healing, yielding an overall healing rate of 100%. The healed tympanic membrane presented a regular morphology, with good graft integration and no obvious retraction, cholesteatoma formation, or recurrent perforation. The audiometric parameters at all postoperative time points were significantly better compared with those before surgery (P<0.05), and continued to improve over time (Table-II). At six months postoperatively, the mean ACT decreased to 25.16±5.38 dB HL, the mean ABG decreased to 8.67±3.15 dB HL, and the hearing success rate reached 94.1% (64/68).

**Table-II:** Comparison of pure tone audiometry results before and after surgery in patients (dB HL).

Time	ACT	BCT	ABG
Preoperative	56.32±8.75	25.07±5.12	31.25±6.42
One month after surgery	38.65±6.21^a^	24.89±4.98	13.76±4.23^a^
Three months after surgery	30.24±5.87^ab^	24.76±4.85	10.48±3.67^ab^
Six months after surgery	25.16±5.38^abc^	24.68±4.72	8.67±3.15^abc^
F	614.9	0.420	598.3
P	<0.001	0.740	<0.001

***Note:*** Compared with preoperative,

a P<0.05; Compared with one month after surgery,

^b^P<0.05; Compared with three months after surgery, ^c^P<0.05.

Subgroup analysis revealed differences in hearing improvement outcomes among patients with varying degrees of calcification. At six months postoperatively, statistically significant differences in mean ACT and ABG were observed among subgroups (P<0.001). This indicated that patients with Grade-III calcification had the worst recovery of air conduction hearing (with the highest ACT value). As shown in Table-III, no significant difference was found between Grade-I and Grade-II AMLC, while Grade-I was significantly superior to Grade-III (P<0.028). For postoperative ABG values, significant differences were detected among all three groups, showing a graded pattern of Grade-I < Grade-II < Grade-III; in other words, higher calcification grades were associated with larger residual ABG values and poorer hearing recovery. For each increase in the grade of calcification, the mean postoperative ABG increased by approximately 3–4 dB HL.

**Table-III:** Comparison of Pure Tone Audiometry Results at six Months Postoperatively Among Patients with Different Degrees of AMLC (dB HL).

Comparison Group	Mean Difference (dB)	Standard Error	q Value	P Value
Post Hoc Pairwise Comparisons of ACT	—	—	—	—
Grade-I vs Grade-II	-3.82	1.359	2.81	0.067
Grade-I vs Grade-III	-5.68	1.702	3.34	0.028
Grade-II vs Grade-III	-1.86	1.568	1.19	0.648
Post Hoc Pairwise Comparisons of ABG	—	—	—	—
Grade-I vs Grade-II	-2.74	0.704	3.89	0.008
Grade-I vs Grade-III	-7.33	0.883	8.30	<0.001
Grade-II vs Grade-III	-4.59	0.813	5.64	<0.001

No severe postoperative complications occurred in any of the patients. Mild otorrhea was observed in two cases (2.9%) within one week postoperatively, which resolved completely after local dressing changes. A sensation of ear fullness was reported in one case (1.5%) at three months postoperatively; tympanometry revealed a type C tympanogram, suggesting transient eustachian tube dysfunction. This symptom was not treated with specific interventions and resolved spontaneously at six months postoperatively. No complications such as facial paralysis, sensorineural hearing loss, vertigo, or cholesteatoma formation were observed during the follow-up period.

## DISCUSSION

This study retrospectively analyzed 68 patients with CHL diagnosed intraoperatively with calcification of AML. All patients underwent endoscopic removal of calcified foci combined with tympanoplasty. The results showed that the primary tympanic membrane healing rate reached 100%. At six months postoperatively, the mean air conduction (AC) threshold decreased to (25.16±5.38) dB HL, the ABG narrowed to (8.67±3.15) dB HL, and the hearing success rate was 94.1%, with no severe complications observed. These findings fully confirm the efficacy and safety of this surgical approach.

As a key structure maintaining the mechanical conduction of the ossicular chain, calcification of the AML leads to decreased elasticity and restricted malleolar mobility, which constitutes an important pathogenic mechanism of CHL. This is consistent with the research conclusion of Huber et al.^3^, who found that fixation of the AML significantly impairs the conduction efficiency of the ossicular chain, and hearing is markedly improved after intraoperative release of calcified tissues. Through further analysis of calcification grading, this study verified a negative correlation between the degree of calcification and hearing prognosis: the postoperative ABG in patients with Grade-III calcification (13.56±3.87 dB HL) was significantly higher than that in patients with Grade-I (6.23±2.15 dB HL) and Grade-II calcification. This finding indicates that the surgical improvement effect is limited when calcification involves the surrounding bone tissue, thus filling the knowledge gap in previous studies regarding the impact of calcification severity.

In terms of diagnosis, the detection rate of calcified foci by high-resolution computed tomography (HRCT) of the temporal bone in this study was only 61.8%, which is far lower than the 100% detection rate by intraoperative endoscopy. This is in line with the results of Fujiwara et al.^10^ who proposed that density changes caused by early ligament calcification are easily masked by the volume effect, while otoscopy can directly observe the morphology and mobility of the ligament and thus serves as the gold standard for diagnosis. In addition, although Tang et al.^11^ improved the detection sensitivity of calcified foci through machine learning-assisted ultra-high-resolution CT analysis, this method still cannot completely replace intraoperative direct visualization, suggesting that clinical practice should attach great importance to the core role of otoscopy in intraoperative evaluation.

Regarding therapeutic efficacy, the 94.1% hearing success rate at six months postoperatively in this study is consistent with the 94.3% hearing improvement rate of traditional endoscopic tympanoplasty reported by Li et al.^12^, Sooriyamoorthy et al.^6^ pointed out that neglect of subtle structural lesions (such as, ligament calcification) is the main cause of poor surgical outcomes in patients with CHL and an intact ossicular chain. In this study, precise management of calcified foci significantly improved the prognostic level. Only 2.9% of patients in this study developed mild external auditory canal oozing, and 1.5% had transient eustachian tube dysfunction. The complication rate was much lower than the 12.8% (10/78) reported by Yao et al.^13^, reflecting the advantages of minimally invasive techniques in protecting the structural integrity of the middle ear.

The etiology of AMLC has not been fully elucidated. In this study, 76.5% of patients were complicated with chronic suppurative otitis media, suggesting that chronic inflammation may be the main risk factor. This is consistent with the mechanism proposed by Khavarghazalani et al.^14^ that inflammatory mediators promote calcium salt deposition. In addition, factors such as trauma and age-related degenerative changes may also be involved in the pathogenesis, which is in agreement with the phenomenon observed by Maillot et al.^15^ that the incidence of ligament calcification is increased in patients with traumatic tympanic membrane perforation.

The core findings of this study confirmed that for patients with confirmed significant calcification of the AML by preoperative HRCT or intraoperative direct visualization, meticulous debridement of the AMLC during endoscopic tympanoplasty was associated with favorable short-term hearing improvement. The underlying mechanisms may include the following three aspects: 1) Load reduction effect: Removal of calcified/ossified hard tissues directly relieves the additional mass burden on the malleus, especially the malleolar neck. 2) Stiffness release effect: Relieving the rigid restraint of calcified ligaments on the rotational movement of the malleus (mainly in the anteroposterior direction) significantly reduces its rotational stiffness and restores its physiological vibrational freedom. 3) Vibration mode optimization: Making the malleus-tympanic membrane complex more sensitive to incoming acoustic waves and more consistent with its inherent rotational vibration mode, thereby improving the sound energy transmission efficiency in the middle and low frequency ranges.

This study can reasonably support the statement that patients with intraoperatively identified AMLC experienced significant short-term hearing improvement after combined management. It may also suggest that AMLC is an under recognized contributing factor in a subset of patients with CHL. However, it does not yet prove that AMLC is an independent and primary cause, nor that all similar patients should routinely undergo this specific intervention.

### Surgical Techniques and Key Considerations:

*Techniques for calcification removal:* For mild calcification, gentle dissection with a micro dissector is feasible; for moderate calcification, gradual curettage with a small curette is required; for severe calcification (especially bony fusion), grinding with a low-speed diamond burr is recommended to avoid thermal injury caused by high-speed rotation. 1-3 Intraoperative malleolar mobility should be repeatedly tested with a probe to ensure complete removal of calcified tissues.

*Selection of tympanic membrane repair materials:* Tragal perichondrium alone may be used for small perforations (<3 mm), whereas a tragal cartilage-perichondrium composite graft is more suitable for larger perforations (≥3 mm) because of its stronger support and resistance to retraction..^16^,^17^

*Protection of surrounding structures:* During the operation, attention should be paid to avoiding the chorda tympani nerve (located below the malleolar manubrium), the horizontal segment of the facial nerve (located above the stapes) and the eustachian tube orifice to prevent iatrogenic injuries.^18^

*Postoperative care:* The packing material in the external auditory canal should be appropriately tight; over-tight packing may affect the blood supply of the tympanic membrane, while loose packing is prone to displacement of the repair material. Forceful nose blowing or water entry into the external auditory canal should be avoided within one month postoperatively to prevent infection.^19^,^20^

### Limitations:

Firstly, the single-center retrospective design with a relatively limited sample size may lead to selection bias. Also, the effects of confounders, such as middle ear mucosal status, eustachian tube function, subtle ossicular stiffness not recognized intraoperatively, and the severity of chronic inflammation, could not be fully excluded in the present study. Secondly, only the overall hearing success rate was reported, and it was not stratified according to AMLC grade, perforation characteristics, or CT status. Without such subgroup analysis, the generalizability of the results to other different scenarios may be limited. Thirdly, in this cohort of patients with intraoperatively identified AMLC, the combined procedure was associated with favorable short-term hearing improvement, but the independent contribution of AMLC removal cannot be isolated within the present design. To strengthen this argument in future work, a comparator group or at least a matched historical cohort undergoing tympanoplasty alone would be highly valuable. Fourthly, patients with conductive hearing loss may be concerned not only with the magnitude of the reduction in the ABG, but also with whether daily communication has improved, whether any sensation of aural fullness has diminished, whether their tinnitus status has changed, whether their subjective auditory perception has been enhanced, and whether any new symptoms have emerged postoperatively. However, such subjective symptoms were not analyzed. Lastly, the lack of long-term follow-up data may result in insufficient assessment of the risk of calcification recurrence, thus a long-term follow-up protocol with an extended observation period of two to five years is needed.

## CONCLUSION

Calcification of the AML is one of the important etiologies of CHL. Its diagnosis primarily relies on intraoperative endoscopic observation, while temporal bone CT can serve as an auxiliary modality. Endoscopic removal of calcified lesions combined with tympanoplasty was associated with favorable short-term hearing improvement, with the advantages of minimal invasiveness, definite curative effect, and few complications. For patients with CHL and intact ossicular chains, the possibility of AMLC should be carefully considered, and appropriate intraoperative management of confirmed AMLC may contribute to favorable hearing outcomes. However, these research results mainly reflect short-term prognosis, and the diagnosis largely depends on intraoperative identification. Therefore, these results should be interpreted with caution.

### Author’s contributions:

**LY:** Literature search, study design and manuscript writing. **JL, YD** and **SS:** data collection, data analysis and interpretation. Critical Review. **LY:** Manuscript revision and validation and is responsible for the integrity of the study. All authors have read and approved the final manuscript.

## References

[1] Fernandez IJ, Rondini F, Presutti L, Molinari G (2023). Recurrence of conductive hearing loss after stapes surgery: a narrative review. Acta Otorhinolaryngol Ital.

[2] Esser J, Lüers JC (2025). Differential Diagnosis of Conductive Hearing Loss. Laryngorhinootologie.

[3] Huber A, Koike T, Wada H, Nandapalan V, Fisch U (2003). Fixation of the anterior mallear ligament: diagnosis and consequences for hearing results in stapes surgery. Ann Otol Rhinol Laryngol.

[4] Batts S, Pham N, Tearney G, Stankovic KM (2025). The State of High-Resolution Imaging of the Human Inner Ear: A Look Into the Black Box. Adv Sci (Weinh).

[5] Ali S, Ghayas Khan MS, Zahid L (2022). Common causes of hearing impairment among children less than 8 years of age in District Bahawalpur, Punjab Pakistan. J Pak Med Assoc.

[6] Sooriyamoorthy T, De Jesus O (2025). Conductive Hearing Loss. In: StatPearls.

[7] Akyigit A, Sakallıoglu O, Karlidag T (2017). Endoscopic tympanoplasty. J Otol.

[8] Kim HJ, Jung HS, Kwak HH, Shim KS, Hu KS, Park HD (2004). The discomallear ligament and the anterior ligament of malleus: an anatomic study in human adults and fetuses. Surg Radiol Anat.

[9] Mattingly JK, Uhler KM, Cass SP (2016). Air-Bone Gaps Contribute to Functional Hearing Preservation in Cochlear Implantation. Otol Neurotol.

[10] Fujiwara M, Watanabe Y, Kashiwagi N, Ohta Y, Sato T, Nishigaki M (2021). Improved visualization of the chorda tympani nerve using ultra-high-resolution computed tomography. Acta Radiol Open.

[11] Tang R, Li J, Zhao P, Zhang Z, Yin H, Ding H (2024). Utility of machine learning for identifying stapes fixation on ultra-high-resolution CT. Jpn J Radiol.

[12] Li P, Zhang Y, Fu QY, Meng QX, Xie JH, Liang Y (2014). The effectiveness of endoscopic tragus cartilage perichondrium myringoplastyin the treatment of large tympanic membrane perforations. J Clin Otorhinolaryngol Head Neck Surg.

[13] Yao ZX, Shao YD, Xiao CQ, Zhang YL, Wang H, Li W (2025). Short-term efficacy of endoscopic repair of tragal cartilage with perichondrium membrane. Chin J of Endoscopy.

[14] Khavarghazalani B, Emadi M, Heidari A, Nahrani MH, Amini S (2025). Neurobiological insights into central auditory processing dysfunction following early-life otitis media. Brain Res Bull.

[15] Maillot O, Attyé A, Boyer E, Heck O, Kastler A, Grand S (2016). Post traumatic deafness: a pictorial review of CT and MRI findings. Insights Imaging.

[16] Wahid FI, Nagra SR (2018). Incidence and characteristics of Traumatic Tympanic Membrane perforation. Pak J Med Sci.

[17] Jiang L, Cherif C, Wöltje M (2026). Fibrous Biomaterial Scaffold for Tympanic Membrane Repair: Microarchitectural Engineering and Structure Function Performance. J Funct Biomater.

[18] Brar S, Watters C, Winters R (2025). Tympanoplasty 2023.

[19] Wahid FI, Saleem M, Muhammad R, Khan MR (2021). Aftermath of traumatic tympanic membrane perforation: Our findings at a tertiary care hospital in Pakistan. Pak J Med Sci.

[20] Ye X, Wang X, Li R, Liu X (2025). Imaging Examination of a Three-Year Follow-up of Cartilage Underlay Myringoplasty with Preserving Perforation Margins for Repairing Small- to Medium-Size Perforation. J Otolaryngol Head Neck Surg.

